# Evaluation of Knowledge and Practice of Pharmacy Professionals regarding the Risk of Medication Use during Pregnancy in Dessie Town, Northeast Ethiopia: A Cross-Sectional Study

**DOI:** 10.1155/2019/2186841

**Published:** 2019-07-25

**Authors:** Abdu Tuha, Yilak Gurbie, Haftom Gebregergs Hailu

**Affiliations:** ^1^Department of Pharmacy, College of Medicine and Health Sciences, Wollo University, P.O. Box 1145, Dessie, Ethiopia; ^2^School of Pharmacy, College Health Sciences, Mekelle University, Mekelle, Ethiopia

## Abstract

**Background:**

The developing organism is unique in its responsiveness to drugs and predictability of therapeutic effectiveness based on the adult which can lead to grave consequences in the neonate and child. Moreover, uncertainty about the risks of drug use in pregnancy could result in restrictive attitudes towards prescribing and dispensing medicines and their use. Pharmacists have huge duties to improve medication use, especially among pregnant women. The objective of this study is, hence, to assess the knowledge and practice of pharmacy professionals (PPs) towards the risk of medication use during pregnancy.

**Methodology:**

A questionnaire-based cross-sectional study was carried out over practicing community and hospital pharmacy professionals in Dessie town. They were asked about the safety of common drugs during pregnancy. It involves both prescription-only medications (POM) and over-the-counter (OTC) medications. Secondly, they were asked about their practice towards the risk of medication use during pregnancy. Both descriptive and analytical statistics were utilized. For descriptive analysis, results were expressed as numbers, percentages, and mean (± SD and 95% CI).

**Result:**

Seventy-six pharmacy professionals in Dessie, Northeast Ethiopia, took part in the study. Most of the respondents (64.5%) believed that amoxicillin is safe in all trimesters. 26 (34.2%) of participants knew that isotretinoin is unsafe for use by pregnant women. About dietary supplements, 32.9% of PPs reported that Vitamin A supplements are safe in all trimesters. There was a significant difference observed for study college and years of experience of the PPs in their score of knowledge test (p=0.020 and p=0.024, respectively). Additionally, there was a difference seen for gender (p=0.030), study college (p=0.036), and working institution (p=0.013) in their advice to pregnant women.

**Conclusion and Recommendation:**

Overall, PPs exhibited very low knowledge about drug safety during pregnancy. The absence of obligatory continuing pharmacy education for pharmacists is expected to have negatively affected the level of medication knowledge and consequently the pharmaceutical care services delivered in community and hospital pharmacies. As medication knowledge of PPs is poor, a multitude of strategies (educational, economic, managerial, and regulatory) should be designed by the government, universities, and pharmaceutical associations to improve the pharmacy professionals' role in the healthcare system by providing them with continuous and up-to-date medication knowledge.

## 1. Background

Drug therapy in pregnant women cannot be completely avoided because some pregnant women may have acute or chronic diseases. Approximately 8 of 10 women reported the use of at least one medication, either prescribed or OTC, during the course of their pregnancy [1]. The developing organism is unique in its responsiveness to drugs and predictability of therapeutic effectiveness based on the adult which can lead to grave consequences in the neonate and child. It should be emphasized that fetal adverse drug effects are not always manifested immediately as in the case of maternal thalidomide ingestion. It is important to note that fetal abnormalities can occur after several months as seen with clonidine or in the case of diethylstilbestrol vaginal adenocarcinoma they can take 20 years to develop. Further ingestion of over-the-counter (OTC) preparations should be limited and deemed to be used with caution. Folate-sensitive neural tube defects (NTDs) are an important, preventable cause of morbidity and mortality worldwide that can be caused by the use of some medications during pregnancy [2]. It is generally accepted that the pregnant mother provides a fetus with an environment in which to develop. However, drug exposure in utero is far more deleterious than in the growing child as the fetus lacks the ability to cope with pharmaceutical agents entering its biosphere [3].

Even for drugs with teratogenic effects, the vast majority of pregnancies with drug exposure will result in normal offspring [4]. Drug dose, route of administration, duration of treatment, and gestational timing are all determinants for teratogenic risk at drug exposure [5]. A drug may be safe at one dosage but may give teratogenic effects if the dose is increased above a threshold level. Systemic drug exposure is also related to the route of administration. For example, dermal administration will reduce the risk of teratogenic effects due to limited systemic absorption. For drugs with potential for teratogenic effects throughout pregnancy, increased duration of treatment may increase the risks for fetal defects [6–9].

A better balance is needed between the risk and benefit of drug treatments during pregnancy. Of course, we have to do our best to reduce the risk of teratogenic drugs as much as possible; however, it is worth stressing the preventive effect of drugs for maternal diseases (e.g., diabetes mellitus and hyperthermia) related congenital abnormalities [10]. To manage the complications associated with pregnancy and motherhood, many medicines are employed. Antibiotics remain important in pregnancy and may be second to only iron and food supplement [11–13]. One of the physicians' roles is to guide patients in weighing risk and benefits, based on available knowledge. The fact that there is scientific uncertainty regarding teratogenic risks of drug use in pregnancy may, however, increase physicians' own perception of risk [14].

Community pharmacists have an important role in selecting appropriate medicines and encouraging good health behaviors. Enhancement of pharmacists' knowledge about treatment in pregnancy is needed and will enhance pharmacists' role in improving maternal health. There is an urgent need to stress the importance of continuous pharmacy education tailored to meet the requirements of specialized areas. Pharmacist should be aware of medications used during pregnancy and should be familiar with risks and benefits of the medication [15–19].

A cross-sectional survey conducted in Tanzania in 2011 [15] to assess the knowledge of drug dispensers and pregnant women regarding drug use in pregnancy demonstrated that good proportion of the study sample lacks adequate knowledge regarding the harmful effects of drugs during pregnancy.

A cross-sectional study that was conducted in Qatar in 2016 indicated that 86% of the respondents were aware of the risk and benefits associated with the medication use in pregnancy. Majority (64.7%) of the pharmacists possessed average knowledge levels and 34.3% had good knowledge. Respondents with experience of 5 years and above had better knowledge levels than others. There was a significant positive association between respondents having continuous educational activities and their knowledge levels (P < 0.001) [20].

An observational cross-sectional study conducted in Beirut, Lebanon, showed that pharmacists with experience of more than 10 years had the highest percentage [21].

In 2006, a cross-sectional study that aimed to assess knowledge and attitudes of pharmacists on dispensing drugs to pregnant women in Curitiba (Brazil) suggested that pharmacists dispensing drugs were not able to interpret information on the use of drugs by pregnant women, and they did not have reliable information sources on the use of drugs during pregnancy. However, they advised and counseled drugs to pregnant women and discussed physicians' therapeutic strategies [22].

A study was conducted in Saudi Arabia to assess medication use, knowledge, and beliefs about medications among pregnant women in 2013. The study revealed that most pregnant women had a positive attitude towards medications in general but they believed pregnant women should be more cautious about drug use during pregnancy. A significant association was found between participants' education and occupation and beliefs about medications. Women indicated inadequate provision of drug-related information from physician and pharmacist; they rely on medication pamphlet to get such information. Most pregnant women (59.2%) were able to identify drugs to be avoided in pregnancy that agreed roughly with FDA categories with 23 hits out of 32 [23].

## 2. Methods

### 2.1. Study Area, Design, and Period

The study was conducted in Dessie town, which is located in the northeast of Ethiopia. Dessie city has five hospitals, twenty-seven pharmacies, and twenty-four drug stores. A questionnaire-based prospective cross-sectional study design was conducted from May 18 to May 27, 2018.

### 2.2. Population and Eligibility Criteria

All community and hospital pharmacists and druggists in Dessie town were taken as study populations. Pharmacy professionals whose working experience is less than 6 months were illegible and excluded.

### 2.3. Sample Size Determination and Sampling Technique

Since the number of study population is small, all pharmacy professionals that meet the inclusion criteria were included.

### 2.4. Data Collection Instrument

Standard instruments were designed to collect information on each of the key components of the study: checklist was used to assess the knowledge levels of drug dispensers in the community and hospital pharmacies regarding dispensing and use of drugs during pregnancy. The checklist contains demographic characteristics of community pharmacists such as age, sex, work experience in the field of pharmacy, the college they graduated from (private or governmental), their latest qualification, working institutions, and their knowledge on nine OTC medications and ten POM. Each question in both sections would only have one of the following answers: safe in the 1st trimester, safe in all trimesters, with weight risks and benefits for individual patients, not safe in the 1st trimester, not safe in all trimesters, and I do not know. The overall knowledge scores were calculated by adding all the correct answers for knowledge questionnaire and the maximum score is 24. Moreover, the questionnaire is made to contain practice questions.

#### 2.4.1. Data Quality Control

To ensure the quality of data, collection tools were standardized. The data collection tool was pretested and modifications were carried out.

### 2.5. Data Analysis and Presentation

Once after all necessary data were obtained, data were checked for completeness, sorted, and categorized accordingly. The data were entered into the SPSS version 20 for windows for analysis. For descriptive analysis, the result was presented using tables, percentages, and mean. Kruskal-Wallis test and Mann-Whitney U test were used to assess intergroup differences. p value of less than 0.05 was considered statistically significant.

## 3. Results

### 3.1. Demographic Characteristics of the Study Participants

A total of 76 PPs out of 120 had participated in the study. The number of participants represents approximately two-thirds of pharmacy professionals. Demographic characteristics of the participants are shown in Table 1.

Sixty percent of the participants were males and the majority (59.2%) had a diploma in pharmacy and the minority (1.3%) had MSc in pharmacy. Sixty-three percent of the participants' ages ranged from 20 to 30 years with an average of 29.4 ± 8.2 years and the average number of years of experience was 5.35. The majority (68.4%) of the participants graduated from governmental institutions of the country. Regarding working institution, the majorities (58%) of participants were working in a private institution and the minority (1.3%) work in both private and governmental institutions. Fifty-five percent were working in community pharmacy.

### 3.2. Knowledge of Pharmacy Professionals of Medication Risk during Pregnancy

Table 2 presents the PPs' response to drug safety during pregnancy. Most of the respondents (64.5%) believed that amoxicillin is safe in all trimesters while 15.8% of respondents believed that it is safe in the first trimester and a small number of participants (2.6%) said that it is used on the basis of risk-benefit assessment. Also, most of PPs (68.4%) said that oral contraceptives are not safe in all trimesters, but very few PPs (6.6%) knew that budesonide is safe in all trimesters. Only 34% of participants knew that isotretinoin is unsafe for use by pregnant women. For central nerves system drugs, about 47.4% of PPs identified phenobarbital as not safe, and approximately one-fifth of PPs correctly identified that lamotrigine should be used only if the potential benefit justifies the potential risk.

Table 3 shows the PPs response to the use of OTC drugs during pregnancy. Among nonprescribed analgesics, the majority of PPs (61.8%) knew that acetaminophen is safe in all trimesters; however, they were in doubt about aspirin usage during pregnancy while 17.1% reported that ibuprofen may be used if the potential benefit justifies the potential risk. A few of the PPs knew that guaifenesin is used only if the potential benefit justifies the potential risk. About dietary supplements, 32.9% of PPs reported that Vitamin A supplements are safe in all trimesters. About 18.4% of the study participants knew that chlorpheniramine could be used after weighing risks and benefits for individual patients.

As shown in Table 4, approximately 53% of the study participants believed that contents of food supplement might be harmful during pregnancy and 39.5% disagreed. About over-the-counter and herbal medicines, the majority (84.2%) of pharmacy professionals agreed to the idea whereas 10.5% disagreed. About the risk of topically applied medicines during pregnancy, 60% of participants recognized that topically applied medicines might be harmful but 22.4% of participants differed. Finally, the study participants were asked about their views regarding US food and drug authority (FDA) pregnancy category risks. Approximately 49% of the study participants decided that FDA pregnancy category “B” is always safer than category “C”. Conversely, 30.3% have disagreed.

Moreover, there was significant difference observed between study college and years of experience of the pharmacy professionals in their score of knowledge test (p=0.020 and p=0.024, respectively) but there was no difference between other variables including gender and working institution (p=0.086 and p= 0.388, respectively) (Table 5).

### 3.3. Practice of Pharmacy Professionals towards Medication Risk during Pregnancy

Ninety-seven of the participants were asking about pregnancy status before dispensing medicines whereas the minority, 2 (2.7%), of the participants were never asking about pregnancy before dispensing. Regarding the practice of PPs when a pregnant woman wants to buy more of the medicine which she is taking, but the PPs know that it is contraindicated during pregnancy, forty-four (57.9%) said that they would advise her to see the doctor again and twenty-two PPs (29%) would advise an alternative medicine which is considered safe in pregnancy (Table 6). Furthermore, there was a difference seen between gender (p=0.030), study college (p=0.036), and working institutions (p=0.013) in their advice to pregnant women.

## 4. Discussion

Medication use during pregnancy is common, and prevalence continues to increase as women's age at pregnancy increases. Pharmacy professionals must carefully appraise the potential risks of medication use versus risks of untreated disease during pregnancy. They should provide patients with information regarding both benefits and risks of medication use while also discussing the limitations of available knowledge so that women are empowered to make informed decisions that are best for them and their babies. Although pharmacists are having great potential to modify and optimize drug therapy in pregnancy, current evidence demonstrates they do not actively provide this care and are least interested in doing so.

This cross-sectional study revealed that PPs have insufficient knowledge about the risk of medications used by pregnant women. This is considered a professional drawback given that pregnant women do consume lots of OTC, POM, and herbal medications. Only 64.5% knew that amoxicillin is safe in all trimesters but very few PPs (6.6%) knew that budesonide is safe in all trimesters. Only 34% of participants knew that isotretinoin is unsafe during pregnancy. This is inconsistent with a study in Saudi Arabia [19] and a study conducted in Palestine [18] which found that about 91% and 82% of CPs correctly identified that isotretinoin is contraindicated during pregnancy, respectively. This study revealed that 68.4% of the study participants knew that oral contraceptives are unsafe in all trimesters comparable to the study in Palestine (75%). About dietary supplements, 32.9% of PPs reported that Vitamin A supplements are safe in all trimesters. About 18.4% of the study participants knew that chlorpheniramine could be used after weighing risks and benefits for individual patients.

There was significant difference observed for study college (p=0.020), in the PPs score of knowledge test, which was closely related to the finding of the research conducted in Saudi Arabia (p=0.015) [19] but inconsistent with a study conducted in Palestine (p=0.94) [18]. The difference is also observed between years of experience of the pharmacy professionals in knowledge score (p=0.024) which was inconsistent with the study in Saudi Arabia (p=0.299). But there was no difference between other variables including gender (consistent with the study in Palestine) and working institution (p=0.086 and p= 0.388, respectively). The study in central Saudi Arabia showed a significant difference between age groups (p=0.001) which is inconsistent with this study (p=0.115).

Ninety-seven percent of the participants in this study were asking about pregnancy status before dispensing medicines whereas the minority, 2 (2.7%), were never asking about pregnancy before dispensing medications. About half of the study participants pointed out that they have sufficient knowledge and 70% were confident about giving advice and counseling to pregnant women. According to a study in Kuwait, in relation to offering advice and solving medication and health problems of pregnant and lactating women, more than half of pharmacists indicated that they have sufficient knowledge (61.5%) and confidence (58.3%), respectively. These drug dispensers were recommending vitamins and food supplements (89.8%) and contraception advice (83.4%), respectively. More than half of the participants indicated that they would recommend medications for headache, constipation, cough, runny nose, sore throat, nausea/vomiting, indigestion, sore or cracked nipple, and insufficient milk. Diarrhea, hemorrhoids, insomnia, varicose vein, swelling of the feet and legs, vaginal itching, back pain, fever, mastitis, and engorgement cases were frequently referred to the physician. Recommendations on medication use were occasionally inappropriate in terms of unneeded drug therapy, off-label use, and safety [24]. A comparative study conducted had revealed that pharmacists' counseling of pregnant women regarding the treatment of common ailments was significantly different between Serbia and Norway in almost all cases [25].

This study revealed that when a pregnant woman is prescribed a contraindicated drug, 57.9% of PPs would advise her to see the doctor again and 29% would advise an alternative medicine which is considered safe in pregnancy. From a study in Thailand [16], it is shown that an equal number of the participants would advise the pregnant woman to see the doctor again and* alternative medicine in this situation*. Furthermore, our study identified that there was a difference seen between gender (p=0.030), study college (p=0.036), and working institution (p=0.013) in their advice to pregnant women. In contrast, the results of the analysis of the study in Thailand indicated that pharmacists' advice for pregnancy had no significant relationship (*χ*2=0.633, p value=0.729) with the gender of pharmacists [16].

The lack of well-designed focused training and the scarce availability of continuing education programs about drugs usage in pregnancy may contribute to the poor knowledge of PPs towards medications safety during pregnancy [2, 26]. Unfortunately and terrifyingly almost three-quarters of the study participants believed that they are confident in giving advice to pregnant women.

### 4.1. Limitation

This study has some limitations. Firstly, the study was conducted in Dessie town and hence may not represent the knowledge and practice of PPs in other regions of Ethiopia. Secondly, apart from using a self-administered questionnaire that introduces bias, it is also a quantitative descriptive study which could not answer the underlying causes of poor knowledge.

### 4.2. Conclusion

Overall, PPs exhibited very low knowledge about drug safety during pregnancy. The absence of obligatory continuing pharmacy education for pharmacists is expected to have negatively affected the level of medication knowledge and consequently the pharmaceutical care services delivered in community and hospital pharmacies. As medication knowledge of PPs is poor, a multitude of strategies (educational, economic, managerial, and regulatory) should be designed by the government, universities, and pharmaceutical association to improve the pharmacy professionals' role in the healthcare system by providing them with continuous and up-to-date medication knowledge.

## Figures and Tables

**Table 1:** Sociodemographic characteristics of the participants.

Variable	*n*(%)
*Gender*	
(i) Male	*46*(60.5)
(ii) Female	*30*(39.5)
*Age*	
(i) 20 to 29	*47*(61.8)
(ii) 30 to 39	*21*(27.6)
(iii) 40 to 49	*7*(9.2)
(iv) 50 to 59	*1*(1.3)
*Marital status*	
(i) Single	*36*(47.4)
(ii) Married	*40*(52.6)
*Level of education *	
(i) Druggist	*45*(59.2)
(ii) Pharmacist	*30*(39.5)
(iii) Msc and Above	*1*(1.3)
*Years of professional experience*	
(i) <5 years	*33*(43.4)
(ii) 5-9 years	*27*(35.5)
(iii) 10-14 years	*15*(19.7)
(iv) >=15 years	*1*(1.3)
*Current working institution*	
(i) governmental	*31*(40.8)
(ii) private	*44*(58.0)
(iii) both	*1*(1.3)
*Current practicing area*	
(i) Hospital Pharmacy	*34*(44.7)
(ii) Community Pharmacy	*42*(55.3)
*Graduation college*	
(i) Private	*24*(31.6)
(ii) Governmental	*52*(68.4)

**Table 2:** Results of medication safety during pregnancy test for pharmacy professionals (POM).

Drug list	Safe in the 1st trimester, *n*(%)	Safe in all trimesters, *n*(%)	With weight risks and benefits for individual patients, *n*(%)	Not safe in the 1st trimester, *n*(%)	Not safe in all trimesters, *n*(%)	I don't know, *n*(%)
Amoxicillin	*12*(15.8)	*49*(64.5)	*2*(2.6)	*5*(6.6)	*6*(7.9)	*2*(2.6)
Ciprofloxacin	*6*(7.9)	*5*(6.6)	*13*(17.1)	*8*(10.5)	*34*(44.7)	*10*(13.1)
Warfarin	*5*(6.6)	*2*(2.6)	*8*(10.5)	*5*(6.6)	*45*(59.2)	*11*(14.5)
Oral Contraceptives	*2*(2.6)	*2*(2.6)	*4*(5.3)	*2*(2.6)	*52*(68.4)	*14*(18.4)
Azithromycin	*6*(7.9)	*30*(39.5)	*12*(15.8)	*10*(13.1)	*10*(13.1)	*8*(10.5)
Phenobarbital	*3*(4.00)	*3*(4.00)	*18*(23.7)	*5*(6.6)	*36*(47.4)	*11*(14.5)
Budesonide, inhaled	*3*(4.00)	*5*(6.6)	*14*(18.4)	*7*(9.2)	*15*(19.7)	*32*(42.1)
Lamotrigine	*0*	*7*(9.2)	*15*(19.7)	*4*(5.3)	*22*(29)	*28*(36.8)
Isotretinoin	*2*(2.6)	*5*(6.6)	*10*(13.1)	*4*(5.3)	*26*(34.2)	*29*(38.2)
Methyldopa	*9*(11.8)	*45*(59.2)	*5*(6.6)	*3*(4)	*3*(4)	*7*(9.2)

**Table 3:** Results of medication safety during pregnancy test for pharmacy professionals (OTC).

Drug list	Safe in the 1st trimester, *n*(%)	Safe in all trimesters, *n*(%)	With weight risks and benefits for individual patients, *n*(%)	Not safe in the 1st trimester, *n*(%)	Not safe in all trimesters, *n*(%)	I don't know, *n*(%)
Acetaminophen	*8*(10.5)	*47*(61.8)	*5*(6.6)	*5*(6.6)	*9*(11.8)	*2*(2.6)
Dextromethorphan	*6*(7.9)	*19*(25)	*15*(19.7)	*9*(11.8)	*13*(17.1)	*14*(18.4)
Guaifenesin	*5*(6.6)	*12*(15.9)	*14*(18.4)	*6*(7.9)	*15*(19.7)	*24*(31.6)
Ibuprofen	*11*(14.5)	*13*(17.1)	*13*(17.1)	*11*(14.5)	*21*(27.6)	*7*(9.2)
Mebendazole	*5*(6.6)	*12*(15.9)	*18*(23.7)	*8*(10.5)	*21*(27.6)	*12*(15.9)
Chlorpheniramine	*8*(10.5)	*8*(10.5)	*14*(18.4)	*9*(11.8)	*19*(25)	*18*(23.7)
Aluminum /magnesium hydroxide	*7*(9.2)	*37*(48.7)	*9*(11.8)	*6*(7.9)	*5*(6.6)	*12*(15.9)
Acetyl salicylic acid	*4*(5.3)	*1*(1.3)	*18*(23.7)	*9*(11.8)	*28*(36.8)	*16*(21.1)
Vitamin A supplement	*10*(13.1)	*25*(32.9)	*6*(7.9)	*3*(4)	*16*(21.1)	*16*(21.1)

**Table 4:** Results of awareness test of PPs about self-care of pregnant women.

Awareness Questions	*n*(%)
*Contents of food supplements might be harmful during pregnancy.*	
(i) Agree	*40*(52.6)
(ii) Disagree	*30*(39.5)
(iii) I don't decide	*6*(7.9)
*Contents of herbal medicine might be harmful during pregnancy.*	
(i) Agree	*64*(84.2)
(ii) Disagree	*8*(10.5)
(iii) I don't decide	*4*(5.3)
*OTC medicines might be harmful during pregnancy.*	
(i) Agree	*64*(84.2)
(ii) Disagree	*8*(10.5)
(iii) I don't decide	*4*(5.3)
*Topically applied medicines might be harmful during pregnancy.*	
(i) Agree	*48*(63.1)
(ii) Disagree	*17*(22.4)
(iii) I don't decide	*11*(14.5)
*A drug which is classified under FDA pregnancy category “B” is always safer than pregnancy category “C”*	
(i) Agree	*37*(48.7)
(ii) Disagree	*23*(30.3)
(iii) I don't decide	*16*(21)

**Table 5:** Comparison of sociodemographic factors with knowledge score and practice of PPs using nonparametric tests (Kruskal-Wallis and Man-Whitney U test).

	Socio-demographic factors and p-value				
	Gender*∗*	Years of Experience*∗∗*	Study College*∗*	Current Practicing Area*∗*	Work Institution*∗*
Knowledge score	0.086	*0.024*	*0.020*	0.533	0.388
Advice to a pregnant women prescribed contraindicated medication	*0.030*	0.674	*0.036*	0.077	*0.013*

*∗*Man-Whitney U test.

*∗∗*Kruskal-Wallis test.

**Table 6:** Practice of pharmacy professionals about contraindicated medicines in pregnancy.

	*n*(%)
*If a pregnant woman wants to buy more of the medicine which she is taking, but you know that it is contraindicated during pregnancy. What advice do you give her?*	
(i) Advise an alternative medicine which is safer	*22*(29)
(ii) Advise to stop this medicine immediately	*10*(13.1)
(iii) Advise to see the doctor again	*44*(57.9)
*I have sufficient knowledge to solve medication and health-related problems of pregnant women*	
(i) Agree	*48*(63.2)
(ii) Disagree	*28*(36.8)
*I am confident about giving advice and counseling to pregnant women.*	
(i) Agree	*70*(92.1)
(ii) Disagree	*6*(7.9)

## Data Availability

The data used to support the findings of this study are available from the corresponding author upon request.

## Abbreviations

CPs: Community pharmacists
FDA: US Food and Drug Authority
NTD: Neural tube defect
OTC: Over-the-counter
POM: Prescription-only medication
PPs: Pharmacy professionals.
